# Comparison of the Characteristics and Prognosis Between Very Young Women and Older Women With Breast Cancer: A Multi-Institutional Report From China

**DOI:** 10.3389/fonc.2022.783487

**Published:** 2022-02-24

**Authors:** Yaping Yang, Weidong Wei, Liang Jin, Haiyan He, Mengna Wei, Shiyu Shen, Hao Pi, Zhiqin Liu, Hengyu Li, Jieqiong Liu

**Affiliations:** ^1^ Guangdong Provincial Key Laboratory of Malignant Tumor Epigenetics and Gene Regulation, Breast Tumor Center, Sun Yat-sen Memorial Hospital, Sun Yat-sen University, Guangzhou, China; ^2^ Department of Breast Surgery, Sun Yat-sen University Cancer Center, Sun Yat-sen University, Guangzhou, China; ^3^ School of Public Health, Tongji Medical College, Huazhong University of Science and Technology, Wuhan, China; ^4^ Department of Thyroid and Breast Surgery, Changhai Hospital, Second Military Medical University, Shanghai, China

**Keywords:** breast cancer, very young, multi-institutional, characteristics and prognosis, comparison

## Abstract

**Purpose:**

Our understanding of breast cancer in very young women (≤35 years old) remains limited. We aimed to assess the clinicopathological characteristics, molecular subtype, and treatment distribution and prognosis of these young patients compared with patients over 35 years.

**Methods:**

We retrospectively analyzed non-metastatic female breast cancer cases treated at three Chinese academic hospitals between January 1, 2008, and December 31, 2018. Local recurrence-free survival (LRFS), disease-free survival (DFS), and overall survival (OS) were compared between different age groups and stratified with distinct molecular subtypes.

**Results:**

A total of 11,671 women were eligible for the final analyses, and 1,207 women (10.3%) were ≤35 years at disease onset. Very young breast cancer women were more likely to be single or childless, have higher-grade disease, have more probability of lymphovascular invasion (LVI) in tumor and triple-negative subtype, and be treated by lumpectomy, chemotherapy especially more anthracycline- and paclitaxel-based chemotherapy, endocrine therapy plus ovarian function suppression (OFS), anti-HER2 therapy, and/or radiotherapy than older women (*P* < 0.05 for all). Very young women had the lowest 5-year LRFS and DFS among all age groups (*P* < 0.001 for all). When stratified by molecular subtype, very young women had the worst outcomes vs. women from the 35~50-year-old group or those from >50-year-old group for hormone receptor-positive (HR+)/human epidermal growth factor receptor 2-negative (HER2−) subtype, including LRFS, DFS, and OS (*P* < 0.05 for all). In terms of LRFS and DFS, multivariate analyses showed similar results among the different age groups.

**Conclusion:**

Our study demonstrated that very young women with breast cancer had higher-grade tumors, more probability of LVI in tumor, and more triple-negative subtype, when compared with older patients. They had less favorable survival outcomes, especially for patients with the HR+/HER2− subtype.

## Introduction

Although the incidence of breast carcinoma in China is lower compared with that in European countries or America, yet, the newly diagnosed breast cancer cases have been increasing in China, particularly in urban areas (1, 2). Nowadays, in China, the most frequent, newly diagnostic cancer is breast cancer, which is also the fifth cause for cancer-related deaths in women (3). A report from the Chinese Cancer Center showed that China had 3.8 million new patients with malignant tumor in 2014, including new 1.69 million female malignant tumor patients, in which the number of newly diagnosed breast cancer accounted for 16.51% (4). The median age at diagnosis of Chinese female patients with breast cancer is 48 years old, which is 14 years younger than that of breast cancer women (62 years old) reported by the Surveillance Epidemiology and End Results (SEER) database (5).

Young breast cancer is usually defined as patients <40 years at diagnosis (6). Prior studies showed that young breast cancer patients were more likely to have adverse tumor characteristics (for instance, higher grade, higher fraction in tumor proliferation, higher probability in lymph vascular invasion, and hormone receptor negative) and worse prognosis than older patients (7–10). Among these women, there is a special group of patients who have been diagnosed at a very young age (≤35 years old). These very young patients may have special needs and meet more challenges, for example, career break, reproductive barriers, sexual dysfunction, unexpected changes in body image, and psychosocial stress. It is of great importance to address the feature and progress of very young breast cancer for guiding clinical treatment.

Because of the much more younger average age, breast cancer women of very young age account for 6.99%~7.43% among all female cases in China (11, 12); therefore, the incidence is higher than that in the United States (<4%) (13). Very few studies to date have focused on very young breast cancer patients; moreover, these limited studies all had small sample size and no data of tumor molecular subtypes (8, 9, 14, 15). Our study aimed to explore the distribution of clinicopathological features, molecular subtypes, and treatment characteristics in non-metastatic breast cancer women with very young age (≤35 years old) in China and to assess the survival differences between age groups.

## Materials and Methods

### Participants

**Figure 1:**
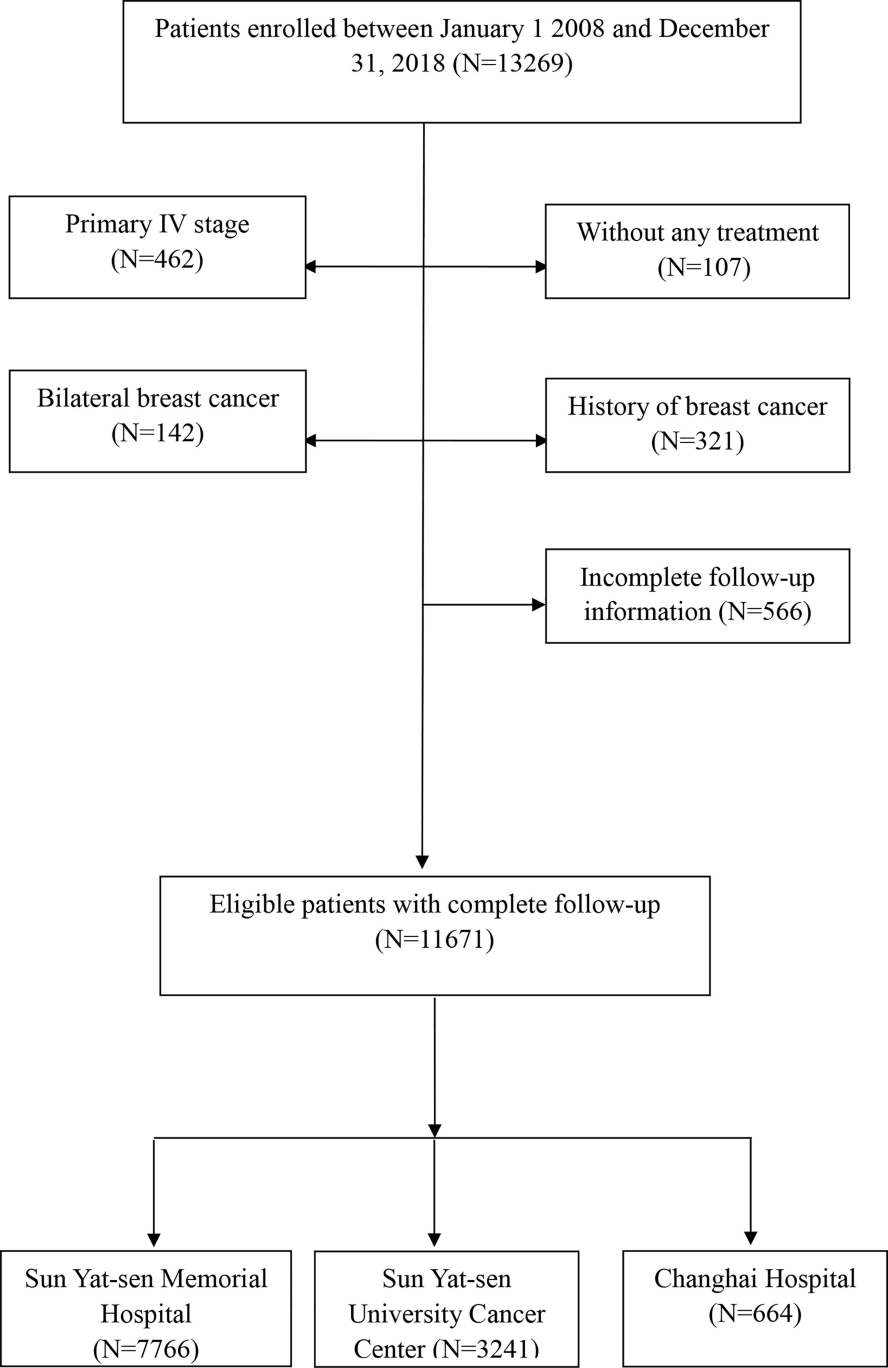
Flowchart of enrollment in the study.

This study reviewed the medical records of all female breast cancer cases who were diagnosed with stage 0 to III according to the American Joint Committee on Cancer Staging Manual (AJCC), 8th edition, and treated between January 1, 2008, and December 31, 2018 at three hospitals, retrospectively. A total of 11,671 women with complete follow-up were included in the current study, consisting of 7,766 patients treated at Sun Yat-sen Memorial Hospital (SYSMH) from Guangzhou, 3,241 patients treated at Sun Yat-sen University Cancer Center from Guangzhou, and 664 patients from Changhai Hospital at Shanghai (**Figure 1**). Patients with previous malignancies in 5 years, history of breast cancer, and stage IV or bilateral breast cancer and who did not receive any treatment were excluded. Approximately 10.3% (1,207/11,671) female breast cancer patients were ≤35 years old during this period of time. The demographic and clinicopathological data and treatment variables were collected from a multicenter online database, “Yixian Database” (16), with strict privacy standards. Survival data were obtained from the follow-up registry of each center and follow-up data were censored on January 31, 2021. This study was approved by Sun Yat-sen Memorial Hospital (SYSMH) Ethics Committee. As this is a retrospective study, informed consent from the study participants can be exempted, and a participant who was newly diagnosed to have breast cancer enrolled into the online “Yixian Database” is a default option at the time of diagnosis.

### Variable Definitions

Clinicopathological features and treatment variables were compared between age groups: ≤35-year-old group, 35~50-year-old group, and >50-year-old group. The information collected included family history, marriage status, reproductive history, histological type, breast surgery, axillary surgery, tumor grade, tumor–node–metastasis (TNM) stage, molecular subtype, and estrogen receptor (ER), progesterone receptor (PR), and human epidermal growth factor receptor 2 (HER2) statuses. Adjuvant therapies included chemotherapy, anti-HER2 therapy, endocrine therapy, and radiation therapy.

### Definition of Survival Endpoints

Local recurrence-free survival (LRFS) was defined as the time from the date of diagnosis and first event of local invasive breast recurrence and regional invasive recurrence. Disease-free survival (DFS) was defined as the time between diagnosis and tumor first recurrence or metastasis or death from any cause. Overall survival (OS) was defined as the time between diagnosis and death from any cause.

### Statistical Analysis

Descriptive statistics were reported as frequency with percentage for categorical variables, and these variables were compared with chi-square tests or Fisher’s exact test according to age groups (≤35 years, 35~50 years, and >50 years). Survival analyses (LRFS, DFS, and OS) were performed with the Kaplan–Meier method and compared using the log-rank test. Univariate Cox regression was analyzed for LRFS, DFS, and OS, respectively. The significant variables in univariate Cox regression were then included in the multivariate Cox regression analyses, and the forward conditional method was used to select independent survival factors. It was two sided in all statistical tests and *P <*0.05 was considered statistically significant. All the statistical analysis of this study was performed with STATA 13.0 (StataCorp, College Station, TX, USA).

## Results

### Baseline Clinicopathological Data

A total of 11,671 (median age: 48 years old; range: 17~95) women with breast cancer were included. Characteristic features and treatment characteristics are outlined in **Table 1**. Among these patients, 1,207 women (10.3%, median age: 32 years) were in the ≤35-year-old group, 5,675 women (48.6%, median age: 44 years) were in the 35~50-year-old group, and 4,789 women (41.0%, median age: 58 years) were in the over 50-year-old group. Overall, the median of follow-up time was 52 months in this study. Compared with older women, very young breast cancer women were more likely to be single and childless (*P* < 0.001) when they were diagnosed. Very young patients had more high-grade disease, more probability of lymphovascular invasion (LVI) in tumor, and more triple-negative subtype (*P* < 0.05). Moreover, very young patients were more likely to receive lumpectomy, chemotherapy especially more anthracycline- and paclitaxel-based chemotherapy, endocrine therapy plus ovarian function suppression (OFS), anti-HER2 therapy, or radiotherapy (*P* < 0.05) (**Table 1**). Women in the ≤35-year-old group had the highest proportion (43.7%) of OFS in HR-positive patients than the other age groups (**Table 1**).

**Table 1:** Descriptive characteristics and treatments of breast cancer patients by age at diagnosis.

		Age group						Total*N* (%)		*P*-value
		≤35 (*n* = 1,207)*N* (%)		35–50 (*n* = 5,675)*N* (%)		>50 (*n* = 4,789)*N* (%)				
Family history of cancer	No	1,165	96.5	5,481	96.6	4,606	96.2	11,252	96.4	0.531
	Yes	42	3.5	194	3.4	183	3.8	419	3.6	
Marital status	Single	146	12.1	143	2.5	108	2.3	397	3.4	<0.001
	Married	1,061	87.9	5,532	97.5	4,681	97.7	11,274	96.6	
Pregnancy	No	265	22.0	468	8.2	305	6.4	1,038	8.9	<0.001
	Yes	942	78.0	5,207	91.8	4,484	93.6	10,633	91.1	
Histological type	IDC	1,007	83.5	4,818	85.0	4,145	86.6	9,970	85.5	<0.001
	ILC	9	0.7	156	2.8	111	2.3	276	2.4	
	DCIS	94	7.8	354	6.2	162	3.4	610	5.2	
	Others	96	8.0	354	6.0	368	7.7	807	6.9	
Tumor grade	1	57	4.7	361	6.4	257	5.4	675	5.8	<0.001
	2	639	52.9	3,134	55.2	2,600	54.3	6,373	54.6	
	3	449	37.2	1,913	33.7	1,760	36.8	4,122	35.3	
	Unknown	62	5.1	267	4.7	172	3.6	501	4.3	
ER	Negative	242	20.1	1,073	19.0	1,076	22.6	2,391	20.6	<0.001
	Positive	962	79.9	4,578	81.0	3,688	77.4	9,228	79.4	
PR	Negative	322	26.7	1,441	25.5	1,683	35.3	3,446	29.7	<0.001
	Positive	882	73.3	4,210	74.5	3,081	64.7	8,173	70.3	
HER2	Negative	903	75.3	4,326	76.6	3,533	74.2	8,762	75.5	0.023
	Positive	297	24.8	1,323	23.4	1,228	25.8	2,848	24.5	
Ki67	<15	309	26.5	1,480	26.8	1,193	25.5	2,982	26.2	0.334
	≥15	857	73.5	4,041	73.2	3,481	74.5	8,379	73.8	
LVI	Negative	914	78.7	4,228	80.3	3,417	82.0	8,559	80.8	0.023
	Positive	247	21.3	1,035	19.7	752	18.0	2,034	19.2	
Molecular subtype	ER+/HER2−	774	64.5	3,783	67.0	3,047	64.0	7,604	65.5	<0.001
	ER+/HER2+	226	18.8	962	17.0	775	16.3	1,963	16.9	
	ER−/HER2+	71	5.9	361	6.4	453	9.5	885	7.6	
	Triple-negative	129	10.8	543	9.6	486	10.2	1,158	10.0	
T stage	0–1	416	34.5	1,837	32.4	1,442	30.1	3,695	31.6	<0.001
	2	523	43.3	2,867	50.5	2,602	54.3	5,992	51.3	
	3–4	166	13.8	668	11.8	594	12.4	1,428	12.2	
	Unknown	102	8.5	303	5.3	151	3.2	556	4.8	
N stage	0	713	59.1	3,429	60.4	2,826	59.0	6,968	59.7	0.721
	1	290	24.0	1,328	23.4	1,135	23.7	2,753	23.6	
	2–3	203	16.8	909	16.0	820	17.1	1,932	16.6	
	Unknown	1	0.1	9	0.2	8	0.2	18	0.2	
Stage	0–1	307	25.4	1,413	24.9	1,095	22.9	2,815	24.1	0.004
	2	582	48.2	2,933	51.7	2,592	54.1	6,107	52.3	
	3	206	17.1	1,009	17.8	942	19.7	2,157	18.5	
	Unknown	112	9.3	320	5.6	160	3.3	592	5.1	
Chemotherapy	Yes	1,101	91.2	5,000	88.1	4,019	83.9	10,120	86.7	<0.001
	No	106	8.8	675	11.9	770	16.1	1,551	13.3	
Chemotherapy treatment	Anthracycline- and paclitaxel-based	709	64.4	2,872	57.4	1,932	48.1	5,513	54.5	<0.001
	Paclitaxel-based	115	10.4	682	13.6	736	18.3	1,533	15.1	
	Anthracycline-based	149	13.5	702	14.0	433	10.8	1,284	12.7	
	Other therapy	29	2.6	161	3.2	142	3.5	332	3.3	
	Unknown	99	9.0	583	11.7	776	19.3	1,458	14.4	
Surgical type	Mastectomy	632	52.4	3,494	61.6	3,441	71.9	7,567	64.8	<0.001
	Breast-conserving surgery	575	47.6	2,181	38.4	1,348	28.1	4,104	35.2	
Nodal surgery	ALND	745	61.7	3,605	63.5	3,364	70.2	7,714	66.1	<0.001
	SLNB	462	38.3	2,070	36.5	1,425	29.8	3,957	33.9	
Endocrine therapy	AI	0	0	180	4.0	2,374	65.6	2,554	28.0	<0.001
	TAM	447	47.1	2,987	65.6	823	22.7	4,257	46.7	
	AI+OFS	140	17.1	429	9.4	25	0.7	616	6.8	
	TAM+OFS	253	26.6	487	10.7	15	0.4	755	8.3	
	Unknown	88	9.3	473	10.4	381	10.5	942	10.3	
Anti-HER2 therapy	No	106	35.9	586	44.8	553	46.3	1,245	44.5	0.006
	Yes	189	64.1	723	55.2	641	53.7	1,553	55.5	
Radiotherapy	No	466	38.6	2,661	46.9	2,781	58.1	5,908	50.6	<0.001
	Yes	741	61.4	3,014	53.1	2,008	41.9	5,763	49.4	

LVI, lymphovascular invasion; IDC, invasive ductal carcinoma; DCIS, ductal carcinoma in situ; ILC, invasive lobular carcinoma; ER, estrogen receptor; HER2, human epidermal growth factor receptor 2; PR, progesterone receptor; SLNB, sentinel lymph node biopsy; ALND, axillary lymph node dissection; OFS, ovarian function suppression; AI, aromatase inhibitor; TAM, tamoxifen.

### Survival Differences Between Age Groups

**Figure 2:**
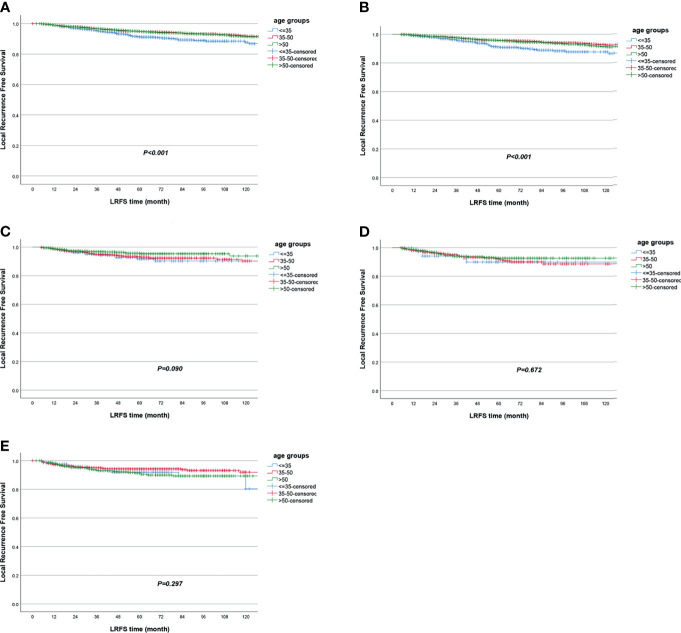
Local recurrence-free survival according to age groups in **(A)** all patients and in subgroup analyses stratified by **(B)** HR+/HER2−, **(C)** HR+/HER2+, **(D)** HR−/HER2+, and **(E)** triple negative.

**Figure 3:**
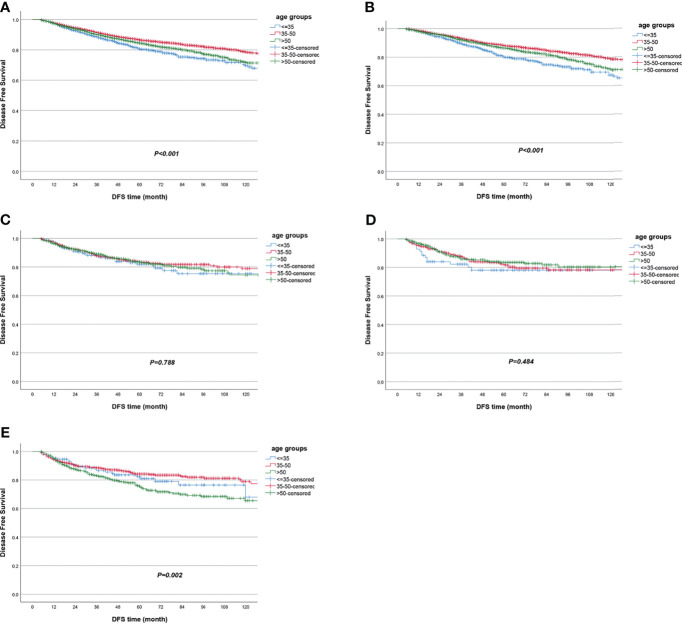
Disease-free survival according to age groups in **(A)** all patients and in subgroup analyses stratified by **(B)** HR+/HER2−, **(C)** HR+/HER2+, **(D)** HR−/HER2+, and **(E)** triple negative.

**Figure 4:**
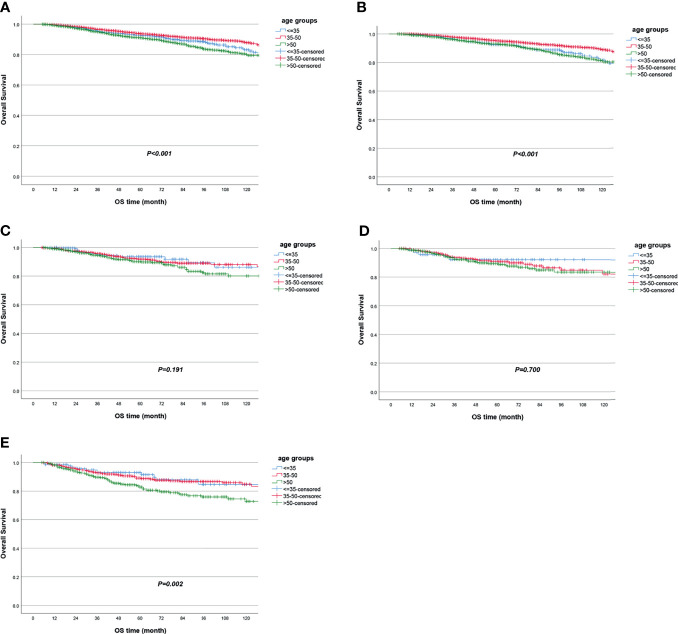
Overall survival according to age groups in **(A)** all patients and in subgroup analyses stratified by **(B)** HR+/HER2−, **(C)** HR+/HER2+, **(D)** HR−/HER2+, and **(E)** triple negative.

The LRFS of all patients was 94.5% [95% confidence interval (95% CI) 94.0%–95.0%] in 5 years. Women in the ≤35-year-old group had the lowest LRFS rate, which was 91.3% (95% CI 89.3%–93.2%) in 5 years. When univariate Cox regression was conducted between age groups, the ≤35-year-old group served as reference, and the HR was 0.63 (95% CI 0.50–0.80, *P* < 0.001) for the 35~50-year-old group and 0.64 (95% CI 0.50–0.82, *P* < 0.001) for the >50-year-old group (**Table 2**, **Figure 2A** and **Supplemental Table 1**). The molecular subtype of HR−/HER2+ had the lowest LRFS rate, which was 92.1% (95% CI 90.0%–94.2%) in 5 years (*P* < 0.001) (**Supplemental Table 1**). The DFS of all patients was 85.0% (95% CI 84.2%–85.8%) in 5 years. Women in the ≤35-year-old group had the lowest DFS rate, which was 80.5% (95% CI 77.8%–83.2%) in 5 years. When univariate Cox regression was conducted between age groups, the ≤35-year-old group served as reference, and the HR was 0.68 (95% CI 0.59–0.79, *P* < 0.001) for the 35~50-year-old group and 0.86 (95% CI 0.74–1.00, *P* = 0.054) for the >50-year-old group (**Table 2** and **Figure 3A**). The OS of all patients was 92.5% (95% CI 91.9%–93.1%) in 5 years. Women in the >50-year-old group had the lowest OS rate, which was 91.0% (95% CI 90.0%–92.0%) in 5 years. When univariate Cox regression was conducted between age groups, the ≤35-year-old group served as reference, and the HR was 0.80 (95% CI 0.64–0.99, *P* = 0.048) for the 35~50-year-old group and 1.30 (95% CI 1.04–1.61, *P* = 0.021) for the >50-year-old group (**Table 2**, **Supplemental Table 1** and **Figure 4A**). Patients with triple-negative breast tumor had the lowest OS rate, which was 86.9% (95% CI 84.7%–89.1%) in 5 years (*P* < 0.001) (**Supplemental Table 1**).

**Table 2:** Analysis of the association between age and survival, adjusted for tumor molecular subtype.

	Age	LRFS			DFS			OS		
		Hazard ratio	95% CI	*P*-value	Hazard ratio	95% CI	*P*-value	Hazard ratio	95% CI	*P*-value
ALL	≤35		Ref			Ref			Ref	
	35~50	0.63	0.50~0.80	<0.001	0.68	0.59~0.79	<0.001	0.80	0.64~0.99	0.048
	>50	0.64	0.50~0.82	<0.001	0.86	0.74~1.00	0.054	1.30	1.04~1.61	0.021
	≤35		Ref			Ref			Ref	
HR+/HER2−	35~50	0.52	0.39~0.71	<0.001	0.60	0.50~0.72	<0.001	0.63	0.48~0.83	0.001
	>50	0.57	0.42~0.78	<0.001	0.78	0.65~0.94	0.010	1.12	0.85~1.46	0.433
	≤35		Ref			Ref			Ref	
HR+/HER2+	35~50	0.91	0.52~1.61	0.747	0.88	0.61~1.27	0.498	1.17	0.66~2.08	0.590
	>50	0.58	0.31~1.08	0.087	0.92	0.63~1.34	0.657	1.52	0.85~2.70	0.156
	≤35		Ref			Ref			Ref	
HR−/HER2+	35~50	0.89	0.37~2.16	0.799	0.77	0.43~1.37	0.370	1.25	0.49~3.21	0.644
	>50	0.73	0.30~1.77	0.482	0.70	0.39~1.26	0.233	1.42	0.56~3.59	0.457
	≤35		Ref			Ref			Ref	
Triple negative	35~50	0.67	0.34~1.34	0.260	0.83	0.53~1.30	0.412	1.17	0.63~2.17	0.618
	>50	0.95	0.48~1.85	0.869	1.36	0.88~2.11	0.172	1.98	1.08~3.63	0.027

HR+, hormone receptor positive; HR−, hormone receptor negative; HER2, human epidermal growth factor receptor 2; LRF, local recurrence-free survival; DFS, disease-free survival; OS, overall survival; 95% CI, 95% confidence interval.

When stratified by tumor molecular subtype, women in the ≤35-year-old group had the worst survival outcomes vs. the 35~50-year-old group and the >50-year-old group for the HR+/HER2− subtype, including LRFS (≤35-year-old group as reference; HR = 0.52, 95% CI 0.39–0.71, *P* < 0.001 for the 35~50-year-old group; HR = 0.57, 95% CI 0.42–0.78, *P* < 0.001 for the >50-year-old group), DFS (≤35-year-old group as reference; HR = 0.60, 95% CI 0.50–0.72, *P* < 0.001 for the 35~50-year-old group; HR = 0.78, 95% CI 0.65–0.94, *P* = 0.010 for the >50-year-old group), and OS (≤35-year-old group as reference; HR = 0.63, 95% CI 0.48–0.83, *P* = 0.001 for the 35~50-year-old group) (**Table 2** and **Figures 2B**, **3B**, **4B**). In the triple-negative subtype, patients in the >50-year-old group also got the worst OS compared with the ≤35-year-old group (HR = 1.98, 95% CI 1.08–3.63, *P* = 0.027) (**Table 2** and **Figure 4E**). In the other molecular subtype, no differences were found between women in the ≤35-year-old group, 35–50-year-old group, and over 50-year-old group (**Table 2** and **Figures 2C–E**, **3C–E**, **4C, D**).

### Univariate and Multivariate Survival Analyses Stratified by Age

Risk factors of survival were selected by univariate analysis. There were several clinicopathological and treatment factors associated with breast cancer survival, including age, family history of cancer, TNM stage, histological type, tumor grade, Ki67, LVI, ER, PR, HER2 status, and receiving of adjuvant therapy (such as endocrine therapy, anti-HER2 therapy, and chemotherapy treatment) (*P* < 0.05) (**Table 3**). Patients with higher stage, higher T/N stage, higher tumor grade, higher Ki67, negative ER/PR status, positive HER2 status, invasive ductal carcinoma (IDC) type, and LVI would get worse survival outcomes (LRFS, DFS, and OS) (*P* < 0.05) (**Table 3**). Moreover, patients who received breast-conserving surgery (BCS), sentinel lymph node biopsy (SLNB), hormone treatment, anti-HER2 therapy, radiotherapy, and chemotherapy would get better survival outcomes (LRFS, DFS, and OS) (*P* < 0.05) (**Table 3**). HER2-positive patients who received anti-HER2 therapy would get better DFS (HR = 0.78, 95% CI 0.64~0.95, *P* = 0.012) and OS (HR = 0.57, 95% CI 0.43~0.76, *P* = 0.012) compared with HER2-positive patients with no anti-HER2 therapy, especially in breast cancer patients diagnosed after 50 years old (DFS: HR = 0.64, *P* = 0.006; OS: HR = 0.51, *P* = 0.002) (**Supplemental Table 2**). However, there were no differences for patients diagnosed ≤35 years old (LRFS: HR = 1.05, *P* = 0.915; DFS: HR = 1.08, *P* = 0.792; OS: HR = 0.51, *P* = 0.159) and 35~50 years old (LRFS: HR = 0.91, *P* = 0.671; DFS: HR = 0.82, *P* = 0.189; OS: HR = 0.68, *P* = 0.068) (**Supplemental Table 2**).

**Table 3:** Univariable survival analyses for age and other clinicopathological factors.

Characters	Subgroups	LRFS			DFS			OS		
		Hazard ratio	95% CI	*P*-value	Hazard ratio	95% CI	*P-*value	Hazard ratio	95% CI	*P*-value
Age group	≤35	Ref			Ref			Ref		
	35~50	0.63	0.50~0.80	<0.001	0.68	0.59~0.79	<0.001	0.80	0.64~1.00	0.048
	>50	0.64	0.50~0.82	<0.001	0.86	0.74~1.00	0.054	1.30	1.04~1.61	0.021
Family history of cancer	Yes vs. no	0.95	0.61~1.48	0.817	0.81	0.61~1.08	0.146	0.72	0.48~1.08	0.115
Marital status	Married vs. single	0.76	0.52~1.12	0.169	0.86	0.68~1.10	0.234	1.01	0.71~1.42	0.977
Pregnancy	Yes vs. no	1.12	0.85~1.49	0.429	1.14	0.96~1.34	0.127	1.32	1.05~1.66	0.018
Surgical type	BCS vs. mastectomy	0.96	0.84~1.14	0.616	0.58	0.52~0.65	<0.001	0.46	0.40~0.54	<0.001
Nodal surgery	SLNB vs. ALND	0.79	0.66~0.95	0.010	0.48	0.43~0.54	<0.001	0.34	0.28~0.411	<0.001
T stage	0–1	Ref			Ref			Ref		
	2	1.39	1.15~1.69	<0.001	1.68	1.49~1.90	<0.001	2.04	1.73~2.42	<0.001
	3	2.11	1.631~2.73	<0.001	3.40	2.94~3.95	<0.001	4.57	3.73~5.59	<0.001
N stage	0	Ref			Ref			Ref		
	1	1.34	1.09~1.63	0.005	1.84	1.63~2.08	<0.001	2.25	1.89~2.66	<0.001
	2–3	2.26	1.86~2.74	<0.001	4.10	3.67~4.59	<0.001	5.50	4.72~6.41	<0.001
Stage	0–1	Ref			Ref			Ref		
	2	1.26	1.01~1.57	0.043	1.72	1.48~2.00	<0.001	2.35	1.86~2.96	<0.001
	3	2.33	1.83~2.96	<0.001	4.91	4.21~5.73	<0.001	7.72	6.12~9.73	<0.001
Histological type	IDC	Ref			Ref			Ref		
	ILC	0.41	0.19~0.92	0.031	1.06	0.78~1.43	0.722	1.02	0.67~1.54	0.936
	DCIS	0.90	0.61~1.32	0.580	0.51	0.38~0.69	<0.001	0.26	0.14~0.45	<0.001
	Others	0.58	0.40~0.85	0.005	0.61	0.49~0.76	<0.001	0.65	0.49~0.85	0.002
Tumor grade	1	Ref			Ref			Ref		
	2	1.17	0.80~1.70	0.419	1.38	1.09~1.734	0.007	1.21	0.89~1.62	0.220
	3	1.61	1.10~2.36	0.014	1.70	1.34~2.15	<0.001	1.64	1.21~2.21	0.001
ER status	Positive vs. negative	0.67	0.56~0.80	<0.001	0.75	0.67~0.83	<0.001	0.62	0.53~0.71	<0.001
PR status	Positive vs. negative	0.65	0.55~0.77	<0.001	0.70	0.63~0.77	<0.001	0.58	0.51~0.67	<0.001
HER2 status	Positive vs. negative	1.28	1.06~1.53	0.009	1.15	1.03~1.28	0.016	1.19	1.02~1.38	0.024
Ki67	≥15 vs. <15	1.69	1.37~2.09	<0.001	1.44	1.28~1.62	<0.001	1.57	1.33~1.85	<0.001
LVI	Positive vs. negative	1.71	1.44~2.09	<0.001	1.68	1.49~1.90	<0.001	1.49	1.25~1.78	<0.001
Hormone treatment	Yes vs. no	0.50	0.35~0.71	<0.001	0.63	0.51~0.79	<0.001	0.66	0.48~0.91	0.010
	TAM	Ref			Ref			Ref		
	AI	0.81	0.63~1.04	0.103	1.07	0.93~1.24	0.359	1.42	1.15~1.74	0.001
Endocrine therapy	AI+OFS	1.71	1.15~2.54	0.008	1.48	1.13~1.93	0.004	1.53	1.00~2.34	0.048
	TAM+OFS	1.59	1.15~2.20	0.005	1.55	1.26~1.91	<0.001	1.19	0.82~1.71	0.361
Anti-HER2 therapy	Yes vs. no	0.85	0.62~1.17	0.330	0.78	0.64~0.95	0.012	0.57	0.43~0.76	<0.001
Radiotherapy	Yes vs. no	1.16	0.99~1.36	0.076	0.99	0.90~1.09	0.850	0.74	0.65~0.84	<0.001
Chemotherapy	Yes vs. no	1.01	0.79~1.30	0.919	0.59	0.49~0.71	<0.001	0.43	0.32~0.57	<0.001
Chemotherapy treatment	Other treatment	Ref			Ref			Ref		
	Anthracycline- and paclitaxel-based	0.86	0.58~1.29	0.478	1.51	1.13~2.02	0.006	1.96	1.27~3.01	0.002
	Paclitaxel-based	0.60	0.37~0.95	0.030	0.88	0.63~1.22	0.441	1.09	0.68~1.77	0.715
	Anthracycline-based	0.75	0.48~1.17	0.206	1.03	0.75~1.42	0.859	1.22	0.77~1.95	0.399

BCS, breast-conserving surgery; IDC, invasive ductal carcinoma; DCIS, ductal carcinoma in situ; ILC, invasive lobular carcinoma; ER, estrogen receptor; HER2, human epidermal growth factor receptor 2; PR, progesterone receptor; LVI, lymphovascular invasion; SLNB, sentinel lymph node biopsy; ALND, axillary lymph node dissection; LRFS, local recurrence-free survival; DFS, disease-free survival; OS, overall survival; 95% CI, 95% confidence interval; AI, aromatase inhibitor; TAM, tamoxifen.

In the multivariable model, clinicopathological characteristics and treatment were controlled for LRFS, DFS, and OS. In the adjusted models, we found that women diagnosed between 35 and 50 years old or diagnosed after 50 years old remained about 35% less likely to develop local recurrence than women diagnosed at ages ≤35 years old (HR = 0.62, 95% CI 0.48–0.81, *P* < 0.001; HR = 0.65, 95% CI 0.50–0.85, *P* = 0.002, respectively) (**Table 4**). For DFS, women diagnosed between 35 and 50 years old were 31% less likely to relapse or develop distant metastasis than those diagnosed at ages ≤35 years old (HR = 0.69, 95% CI 0.59–0.81, *P* < 0.001), and women diagnosed after 50 years old were 20% less likely to relapse or develop distant metastasis than women diagnosed at ages ≤35 years old (HR = 0.80, 95% CI 0.68–0.95, *P* = 0.010) (**Table 4**).

**Table 4:** Multivariable survival analyses stratified by age, adjusted for clinicopathological factors.

		LRFS			DFS			OS		
		Hazard ratio	95% CI	*P*-value	Hazard ratio	95% CI	*P*-value	Hazard ratio	95% CI	*P*-value
Age	≤35	Ref			Ref			Ref		
	35~50	0.62	0.48~0.81	<0.001	0.69	0.59~0.81	<0.001	0.80	0.63~1.03	0.082
	>50	0.65	0.50~0.85	0.002	0.80	0.68~0.95	0.010	1.13	0.88~1.44	0.345
T stage	0–1	Ref			Ref			Ref		
	2	1.23	0.99~1.53	0.060	1.32	1.15~1.51	<0.001	1.48	1.22~1.80	<0.001
	3–4	1.49	1.12~1.99	0.007	2.00	1.69~2.38	<0.001	2.65	2.10~3.34	<0.001
N stage	0	Ref			Ref			Ref		
	1	1.23	0.98~1.54	0.078	1.66	1.45~1.90	<0.001	2.34	1.93~2.83	<0.001
	2–3	1.96	1.55~2.47	<0.001	3.32	2.91~3.78	<0.001	5.87	4.87~7.08	<0.001
LVI	Positive vs. negative	1.35	1.09~1.69	0.007	/	/	/	/	/	/
Ki67	≥15 vs. <15	1.52	1.24~1.91	<0.001	1.31	1.15~1.48	<0.001	1.38	1.15~1.65	<0.001
ER	Positive vs. negative	0.62	0.51~0.75	<0.001	/	/	/	0.81	0.66~1.00	0.048
PR	Positive vs. negative	/	/	/	0.80	0.71~0.89	<0.001	0.77	0.63~0.94	0.009
Surgical type	BCS vs. mastectomy	/	/	/	0.80	0.71~0.91	0.001	/	/	/
Chemotherapy	Yes vs. no	0.68	0.51~0.91	0.010	/	/	/	/	/	/
Radiotherapy	Yes vs. no	/	/	/	/	/	/	0.50	0.43~0.59	<0.001

ER, estrogen receptor; HER2, human epidermal growth factor receptor 2; PR, progesterone receptor; LVI, lymphovascular invasion; BCS, breast-conserving surgery; LRFS, local recurrence-free survival; DFS, disease-free survival; OS, overall survival; 95% CI, 95% confidence interval.

## Discussion

This study demonstrated that very young women (age group ≤35 years) with breast cancer had more higher-grade tumors, more probability of LVI in tumor, and more triple-negative subtype and received more lumpectomy, more chemotherapy especially more anthracycline- and paclitaxel-based chemotherapy, and more endocrine therapy plus OFS, anti-HER2 therapy, and adjuvant radiotherapy when compared with older patients. Moreover, very young women with breast cancer had the lowest 5-year LRFS and DFS among all age groups. When stratified by molecular subtype, very young women with breast cancer had the worst outcomes vs. women in the 35~50-year-old group or those in the >50-year-old group for the HR+/HER2− subtype, including LRFS, DFS, and OS.

The recent European Society of Breast Cancer Specialists (EUSOMA) and the ESO-ESMO fourth international consensus guidelines defined young breast cancer women as those who were diagnosed at or before age 40 (6). Very young breast cancer women were described as those diagnosed at age ≤35 years old, consisting of a unique group of patients that may need further investigation. In this study, very young (≤35 years old) women with breast cancer account for 10.3% of all female breast cancer cases that were diagnosed as stage 0 to III in China; this incidence was similar to the reports from South Korea (9) and Egypt (14), but much higher than the studies from Greece (17) and other European countries (8). This is the largest report from China focusing on very young non-metastatic breast cancer patient cohorts (*n* = 1,207) and, globally, the second largest study so far for these special breast cancer populations. This study reported the clinicopathological characteristics, tumor molecular subtype, and treatment distributions of these special very young onset breast cancer women. Furthermore, the survival differences between age groups, especially stratified by molecular subtype, were explored as well.

We observed that female patients diagnosed with breast cancer at a very young age (≤35 years old) were more likely to be single and childless than older patients, so this group of patients may need to face this life-threatening disease alone and meet more problems, such as stress of establishment of family, inability of childbearing, and the negative impact of distinct treatment on sexuality or body image. They also had more high-grade tumors, which was consistent with prior reports (8, 9, 14). Moreover, we found that very young breast cancer women had more probability of LVI in tumor and more triple-negative disease than older women. For treatment options, we observed that very young breast cancer patients were less willing to receive mastectomy compared with older female patients in China, which was in agreement with a prior Korean study (9). Our study is the only research with available data of chemotherapy, OFS, and adjuvant radiation therapy, and we found that very young patients might receive more chemotherapy especially more anthracycline- and paclitaxel-based chemotherapy, and more endocrine therapy plus OFS, anti-HER2 therapy, and/or adjuvant radiotherapy than older patients. This may be due to the situation that these young women had more aggressive tumors (higher grade, more LVI in tumor, or triple-negative subtype) and preferred breast-conserving surgery because of higher cosmetic requirements.

Not surprisingly, we have found that the younger age group (women ≤35 years old) was an independent risk factor of LRFS and DFS. Moreover, we demonstrated that very young breast cancer women with HR+/HER2− subtype showed the most unfavorable survival outcomes including LRFS, DFS, and OS when compared with older patients. This finding was similar to the results of prior studies which demonstrated that HR+ very young breast cancer women had worse outcomes than older women (7–9). Patients diagnosed after 50 years old had the lowest OS rate and had similar result in patients with triple-negative breast tumor. A study showed that in ER-negative patients, there were no differences between the ≤35-year-old group and the >35-year-old group (8) for OS. Other reports found that triple-negative breast cancer patients ≤50 years of age had worse OS and BCS than the 51–60 age group patients (7). Hence, in patients with triple-negative breast cancer, the effect of age on survival outcomes was unclear, which needs further investigation.

There were several studies reporting that very young breast cancer patients more frequently expressed a high proliferation cell surface marker, and these young patients more frequently had endocrine-resistance features compared with older women (18–21). These findings may partially explain our results of the unfavorable prognosis of HR+/HER− subtype among patients at a very young age. Further studies are required to assess the underlying mechanisms why these younger patients have such molecular profile. The TEXT/SOFT trial demonstrated that compared with tamoxifen alone, OFS plus exemestane or tamoxifen was correlated with much more favorable survival outcomes among HR+ breast cancer women diagnosed at age ≤35 years old (22). However, there were reports that women with younger age had lower adherence and were more likely to give up halfway to endocrine therapy including OFS (23–25). Efforts such as providing social support, establishing good patient–physician relationship, and minimizing the side effects of therapy should be made to address this problem for these very young HR+/HER2− breast cancer patients. This study was based on the multicenter online database “Yixian Database” (5), and 11,671 women with breast cancer were included, which was a large cohort to explore the very young breast cancer patients’ characteristics and prognosis. Because of the higher-grade tumors and more triple-negative subtype for these very young patients, they also had the worst survival outcomes than the other age groups. We should pay more attention to these very young patients with breast cancer.

We acknowledged that there were some limitations in this study. Firstly, as the detailed information of adjuvant endocrine therapy’s treatment duration was not investigated in our study, the findings should be interpreted carefully. Furthermore, hormone receptor and HER2 statuses were used instead of the molecular subtype classification in this study, although this was commonly applied by several population-based studies on breast cancer. The lack of genetic testing results of BRCA1/2 mutations for these very young female breast cancer patients and the lack of family support and economic situation were other limitations.

### Conclusion

In summary, this multi-institutional study demonstrated that very young women with breast cancer in China had more high-grade tumors and more triple-negative subtype and were more likely to receive lumpectomy, neoadjuvant chemotherapy, and radiotherapy than older patients. Moreover, they had less favorable survival outcomes, especially for patients with HR+/HER2− tumors. Further studies are required to focus on this special subpopulation of female breast cancer patients with very young age and to specifically address some clinical characteristics and prognostic outcomes.

## Data Availability Statement

The original contributions presented in the study are included in the article/**Supplementary Material**. Further inquiries can be directed to the corresponding author.

## Ethics Statement

This study was approved by the Ethics Committee of Sun Yat-sen Memorial Hospital, Sun Yat-sen University, with approval number (2015-BA-041). As this was a retrospective study, written informed consent to participate in this study was excused by the institutional review board.

## Author Contributions

JL and YY made main contributions to the study conception and design. YY, HL, WW, HP, HH, SS, ZL, and MW contributed to the data collection, analysis, and interpretation. LJ and JL drafted the article and revised it. All authors agreed to submit the study to the current journal and gave final approval of the version to be published.

## Funding

This work was supported by grants from the National Natural Science Foundation of China (Grant No. 82003311, recipient: YY; 82072906, recipient: JL), Sun Yat-sen Memorial Hospital Cultivation Project for Clinical Research (Grant No. SYS-Q-202004, recipient: YY), Sun Yat-sen Memorial Hospital Yat-sen Scientific Research Launch Project (Grant No. YXQH201920, recipient: YY), Medical Science and Technology Research Fund of Guangdong Province (No. A2020391, recipient: YY), and Guangzhou Science and Technology Program (No. 202102010272, recipient: YY).

## Conflict of Interest

The authors declare that the research was conducted in the absence of any commercial or financial relationships that could be construed as a potential conflict of interest.

## Publisher’s Note

All claims expressed in this article are solely those of the authors and do not necessarily represent those of their affiliated organizations, or those of the publisher, the editors and the reviewers. Any product that may be evaluated in this article, or claim that may be made by its manufacturer, is not guaranteed or endorsed by the publisher.

## Supplementary Material

Supplementary Table 1 Differences of 5 years survival outcomes between age groups and molecular subtypes in breast cancer patients. HR+, Hormone receptor positive; HR-, Hormone receptor negative; HER2, human epidermal growth factor receptor 2; LRFS, Local recurrence free survival; DFS, Disease free survival; OS, Overall survival; 95%CI, 95% confidence interval.

Supplementary Table 2 Analysis of the association between therapy, age and survival for different tumor molecular subtypes. HER2, human epidermal growth factor receptor 2; LRFS, Local recurrence free survival; DFS, Disease free survival; OS, Overall survival; 95%CI, 95% confidence interval.

## Abbreviations

LRFS, local recurrence-free survival; DFS, disease-free survival; OS, overall survival; SEER, Surveillance Epidemiology and End Results database; AJCC, American Joint Committee on Cancer Staging Manual; TNM, tumor–node–metastasis stage; HR+, hormone receptor positive; HR−, hormone receptor negative; ER, estrogen receptor; PR, progesterone receptor; HER2, human epidermal growth factor receptor 2; EUSOMA, European Society of Breast Cancer Specialists; OFS, ovarian function suppression; LVI, lymphovascular invasion; IDC, invasive ductal carcinoma; DCIS, ductal carcinoma *in situ*; ILC, invasive lobular carcinoma; AI, aromatase inhibitor; TAM, tamoxifen; BCS, breast-conserving surgery; SLNB, sentinel lymph node biopsy; ALND, axillary lymph node dissection; 95% CI, 95% confidence interval.
