# Supplementary material for: Comparison of the Characteristics and Prognosis Between Very Young Women and Older Women With Breast Cancer: A Multi-Institutional Report From China
Source: Front Oncol. 2022 Feb 24;12:783487. doi: 10.3389/fonc.2022.783487 (PMC8907474; doi:10.3389/fonc.2022.783487)
Supplement: Supplementary Table 1 — Differences of 5 years survival outcomes between age groups and molecular subtypes in breast cancer patients. HR+, Hormone receptor positive; HR-, Hormone receptor negative; HER2, human epidermal growth factor receptor 2; LRFS, Local recurrence free survival; DFS, Disease free survival; OS, Overall survival; 95%CI, 95% confidence interval. [file Table_1.docx]

Supplemental table 1 Differences of 5 years survival outcomes between age groups and molecular subtypes in breast cancer patients

|  |  |  |  | |  | |
| --- | --- | --- | --- | --- | --- | --- |
| Age group | LRFS(%) | 95%CI | DFS(%) | 95%CI | OS(%) | 95%CI |
| Total | 94.5 | 94.0~95.0 | 85.0 | 84.2~85.8 | 92.5 | 91.9~93.1 |
| <=35 | 91.3 | 89.3~93.2 | 80.5 | 77.8~83.2 | 92.6 | 90.9~94.4 |
| 35-50 | 94.9 | 94.2~95.6 | 86.5 | 85.4~87.5 | 93.7 | 92.9~94.5 |
| >50 | 94.9 | 94.1~95.6 | 84.3 | 83.1~85.6 | 91.0 | 90.0~92.0 |
| HR+ /HER2- | 95.2 | 94.6~95.8 | 86.5 | 85.6~87.4 | 94.0 | 93.4~94.7 |
| HR+ /HER2+ | 93.8 | 92.5~95.1 | 83.2 | 81.2~85.2 | 91.3 | 89.7~92.8 |
| HR- /HER2+ | 92.1 | 90.0~94.2 | 82.5 | 79.6~85.4 | 90.1 | 87.8~92.5 |
| Triple negative | 92.8 | 91.2~94.4 | 80.4 | 77.8~83.0 | 86.9 | 84.7~89.1 |

HR+: Hormone receptor positive; HR-: Hormone receptor negative; HER2, human epidermal growth factor receptor 2; LRFS: Local recurrence free survival, DFS: Disease free survival, OS: Overall survival, 95%CI: 95% confidence interval
