# Supplementary material for: Comparison of the Characteristics and Prognosis Between Very Young Women and Older Women With Breast Cancer: A Multi-Institutional Report From China
Source: Front Oncol. 2022 Feb 24;12:783487. doi: 10.3389/fonc.2022.783487 (PMC8907474; doi:10.3389/fonc.2022.783487)
Supplement: Supplementary Table 2 — Analysis of the association between therapy, age and survival for different tumor molecular subtypes. HER2, human epidermal growth factor receptor 2; LRFS, Local recurrence free survival; DFS, Disease free survival; OS, Overall survival; 95%CI, 95% confidence interval. [file Table_2.docx]

Supplemental table 2 Analysis of the association between therapy, age and survival for different tumor molecular subtypes

|  |  |  | LRFS |  |  | DFS |  |  | OS |  |
| --- | --- | --- | --- | --- | --- | --- | --- | --- | --- | --- |
|  | Age | Hazard ratio | 95%CI | *P* value | Hazard ratio | 95%CI | *P* value | Hazard ratio | 95%CI | *P* value |
| Anti-HER2 therapy  Yes vs No | <=35 | 1.05 | 0.43 ~2.54 | 0.915 | 1.08 | 0.61~1.92 | 0.792 | 0.51 | 0.20~1.30 | 0.159 |
|  | 35~50 | 0.91 | 0.59 ~1.41 | 0.671 | 0.82 | 0.62~1.10 | 0.189 | 0.68 | 0.45~1.03 | 0.068 |
|  | ＞50 | 0.68 | 0.39~1.17 | 0.164 | 0.64 | 0.47~0.88 | 0.006 | 0.51 | 0.34~0.78 | 0.002 |

HER2: human epidermal growth factor receptor 2; LRFS: Local recurrence free survival, DFS: Disease free survival, OS: Overall survival, 95%CI: 95% confidence interval.
